# PCR-based gene synthesis to produce recombinant proteins for crystallization

**DOI:** 10.1186/1472-6750-8-44

**Published:** 2008-04-29

**Authors:** Damien Marsic, Ronny C Hughes, Miranda L Byrne-Steele, Joseph D Ng

**Affiliations:** 1 ExtremoZyme Inc, HudsonAlpha Institute for Biotechnology, 601 Genome Way, Huntsville, AL 35806, USA; 2 Laboratory for Structural Biology, Department of Biological Sciences, University of Alabama, Huntsville, AL 35899, USA

## Abstract

**Background:**

Gene synthesis technologies are an important tool for structural biology projects, allowing increased protein expression through codon optimization and facilitating sequence alterations. Existing methods, however, can be complex and not always reproducible, prompting researchers to use commercial suppliers rather than synthesize genes themselves.

**Results:**

A PCR-based gene synthesis method, referred to as SeqTBIO, is described to efficiently assemble the coding regions of two novel hyperthermophilic proteins, PAZ (Piwi/Argonaute/Zwille) domain, a siRNA-binding domain of an Argonaute protein homologue and a deletion mutant of a family A DNA polymerase (PolA). The gene synthesis procedure is based on sequential assembly such that homogeneous DNA products can be obtained after each synthesis step without extensive manipulation or purification requirements. Coupling the gene synthesis procedure to *in vivo *homologous recombination techniques allows efficient subcloning and site-directed mutagenesis for error correction. The recombinant proteins of PAZ and PolA were subsequently overexpressed in *E. coli *and used for protein crystallization. Crystals of both proteins were obtained and they were suitable for X-ray analysis.

**Conclusion:**

We demonstrate, by using PAZ and PolA as examples, the feasibility of integrating the gene synthesis, error correction and subcloning techniques into a non-automated gene to crystal pipeline such that genes can be designed, synthesized and implemented for recombinant expression and protein crystallization.

## Background

Gene synthesis is a convenient method of obtaining sequence-verified cloned DNA, especially when the source biological material is not readily available (rare, lost or dangerous specimens), when codons need to be optimized for expression in a particular host or when the desired sequence is chimeric or designed *de novo*. In a gene to structure pipeline utilizing X-ray crystallography, customizing the synthesis of the open reading frame to rapidly express recombinant protein for crystallization becomes particularly useful in structure determination endeavors. This is particularly the case when specific residues within a certain site or entire regions of a protein need to be altered to change protein activity, stability or ability to crystallize.

Structural genomics consortia have exploited gene synthesis technology to produce difficult recombinant proteins for structural analysis [1]. In most cases, if not all, synthetic genes are fabricated commercially for convenience and time efficiency. Presently, the affordability of synthetic oligonucleotides (the building blocks for gene synthesis) makes it possible for individual researchers to carry out their own gene synthesis projects in their home laboratories if gene synthesis procedures can be streamlined and performed without intricate manipulations. In addition, combining feasible gene synthesis techniques with quick cloning methods into recombinant expression systems without extensive enzymatic requirements would also greatly facilitate protein structure investigations.

In this study, we use a PCR-based gene synthesis technique coupled to *in vivo *homologous recombination to quickly construct genes for protein production without the use of any additional enzyme. The coding sequences of two proteins derived from hyperthermophilic microorganisms have been synthesized and transformed with a specially prepared expression vector into a bacterial expression system without DNA digestion or ligation. Recombinant proteins were overexpressed from the synthetic gene constructs, purified and used for protein crystallization. Using this method, the entire procedure from gene to crystal screening can be performed within 2 weeks. The procedure described here would especially benefit small traditional structural biology laboratories as the technique to synthesize DNA coding sequences for direct protein production requires no automation and can be executed without great cost and time consumption.

## Methods

### Source of DNA sequences

The nucleotide coding regions of a PAZ (Piwi/Argonaute/Zwille) domain and a deletion mutant (lacking the 5'-3' exonuclease domain) of a family A DNA polymerase (PolA) were selected for synthesis. The sequences of the synthesized genes were derived from the genetic material of two hyperthermophilic microorganisms isolated from mud samples collected at the Rainbow hydrothermal vent field on the North-Atlantic Ridge: the sulfur-reducing archaeon *Thermococcus thioreducens *[2] and an uncharacterized bacterium designated OGL-7B (unpublished). The latter was isolated as a single colony from a 90°C culture of an autoclaved mud sample and could not be cultured further. The PAZ domain and PolA nucleotide sequences, hereafter designated as *paz *and *polA*, were derived from *Thermococcus thioreducens *and OGL-7B respectively.

### Preparation of linearized vector

About 5 μg of pET3a plasmid (Novagen, Madison, USA) was double digested with NdeI and BamH1 restriction endonucleases (Promega, Madison, WI, USA) following the manufacturer's instructions. The digested plasmid was purified using the QIAquick PCR purification kit (Qiagen, Valencia, CA, USA), blunt-ended with Klenow fragment (Promega, Madison, WI, USA) according to the manufacturer's directions and further purified as described above. The resulting pET3a fragment was used for homologous recombination reactions without further modification.

### Design of oligonucleotides for gene synthesis

Oligonucleotides for gene synthesis were designed using DNAWorks [3,4] with the following parameters: *E. coli *class II codon frequency table, 60°C annealing temperature, 60 (for *polA*) and 75 (for *paz*) nt oligonucleotide length, 5 solutions and TBIO mode. Default values were kept for other parameters. For each gene, the input sequence consisted of 3 elements: 30 nt of the pET3a (Novagen, Madison, USA) vector sequence upstream of the NdeI site, followed by the amino acid sequence corresponding to the gene to be synthesized, and finally 30 nt of the pET3a vector sequence downstream of the BamHI site. Solutions with the lowest scores were selected for synthesis. Oligonucleotides were synthesized by Operon (Huntsville, AL, USA) and diluted to a working concentration of 2 μM. Oligonucleotide sequences for *polA *and *paz *are shown in Tables 1 and 2 respectively.

**Table 1:** Oligonucleotides for the synthesis of *polA*

f22	TTTGTTTAACTTTAAGAAGGAGATATACATATGCTCCGCAAGG
f21	ATATACATATGCTCCGCAAGGTCGAGGTACAAACCGTAGAGCCGGAGGACCGTGAAAGCT
f20	GGAGGACCGTGAAAGCTGGAACGACTTCCTGGAAAATCCACTCCTCAGCCTGTGGCTCGA
f19	TCAGCCTGTGGCTCGAGATGGACGGCGAAAACTATCATCGCGCAGAAATCATCGGTCTCG
f18	GCAGAAATCATCGGTCTCGCACTGTCTGATGGTGAGACCCATCTGTACGTCCCTTGGCGC
f17	GTACGTCCCTTGGCGCACTGCGCGTGACTGGGAGAACCTCCACCGTCTGCTCGCGGACGA
f16	TCTGCTCGCGGACGAGGAACGCAAGAAGATTGTTTATGATGGTAAACGTCTGCAAGTGGT
f15	GTAAACGTCTGCAAGTGGTGCTGAAGCGTCGTGGTCTGGAGGCTGGCGGTCTGGCTTTCG
f14	GGCGGTCTGGCTTTCGACGCGCTCCTGGCTTCTTATCTGCTGGACCCGTCCGAATCTGGC
f13	CCCGTCCGAATCTGGCCACTCTCTGTCCGACCTGGTACAGCGTAAGATGGATGGCTCTCT
f12	CGTAAGATGGATGGCTCTCTCCCACCTGATGAGGAGGTTTACGGTAAAGGTGCCAAACGC
f11	GGTAAAGGTGCCAAACGCCGCCTGCCAGGTGAGCGCGAACTCGCTGAACATCTCGCGCGT
f10	CTGAACATCTCGCGCGTAAAGCGGAAGCGCTCAAGCGCCTCTACCCACTGCTGAGCGAGG
f9	CCACTGCTGAGCGAGGAGATCCGTGAGGCCGGTATGGAGAGCCTGCTCTTTGAAATGGAA
f8	GCCTGCTCTTTGAAATGGAACTGCCACTGAGCCGTGTTCTGGCAGAGATGGAGCTGCATG
f7	CAGAGATGGAGCTGCATGGCGTACGTGTCGACCGCGACCGTCTCCTGGACCTGGGTGAAG
f6	CCTGGACCTGGGTGAAGAACTCAAGGAACAGGCAGAATCCCTCACGCGTCAAATCTACGA
f5	TCACGCGTCAAATCTACGAACTGGCGGGTACCGAATTCAACATTAACTCTCCGAAACAGC
f4	ACATTAACTCTCCGAAACAGCTCGCCGAGATTCTCTACGACAAACTGGGTCTGCCGGTTC
f3	CTGGGTCTGCCGGTTCTCAAAAAGACTAAAACGGGCTATTCTACCTCTGCGGACGTTCTG
f2	CCTCTGCGGACGTTCTGGAGAAACTGGCACCGCAACATGAAATCGTTGAAAAAATCCTGC
f1	GAAATCGTTGAAAAAATCCTGCACTACCGCCAAATCATGAAACTGATCTCCACCTATGTC
r1	TTTACCGGATTCCGGGTCGATTTCCTTCAGGAGGCCCTCGACATAGGTGGAGATCAGTTT
r2	GCGGCCAGTTGCAGTGATAGTCTGGTTGAAACGCGTGTGAATTTTACCGGATTCCGGGTC
r3	CAGGCGAATCGGGATATTTTGCAGGTTCGGCTCGGTAGAGCTGAGGCGGCCAGTTGCAGT
r4	CAGACGGAACGAATACCTGACGGATGCGACGACCTTCCTCCAGGCGAATCGGGATATTTT
r5	AATCTGAGAGTAATCTGCGCTCAGGATCTGCCAACCCGGTTCAGACGGAACGAATACCTG
r6	GAGACTCGTCGCCAGACAGATGAGCCAGTACGCGGAGCTCAATCTGAGAGTAATCTGCGC
r7	GTCTTAGTATGAATGTCCATGTCTTCGCTAAAAGCCTGTTTGAGAGACTCGTCGCCAGAC
r8	AACTTCATCTTCAGGGACACCGAACACATCCATAGCGGTCTTAGTATGAATGTCCATGTC
r9	CGAAATTAACCGCCTTTGCTTGACGACGCATGAGAGAGGTAACTTCATCTTCAGGGACAC
r10	GTTTTGGGACAGGCCGTAATCAGAAATGCCGTAGATGATACCGAAATTAACCGCCTTTGC
r11	AACGTTCAATGAATTGCGCCGCTACTTTACGTGGGATGTTGAGGTTTTGGGACAGGCCGT
r12	ACGATCCATGTATTCTTTAACACCAGGGTAAGATTGGAAGTAACGTTCAATGAATTGCGC
r13	TGGTAACGTAACCGTCTTTACGGGCTTGCTCTACAACACGATCCATGTATTCTTTAACAC
r14	AGCGAATTTCCGGGAGGTAGCGACGGCGGTTGAGCATGGTGGTAACGTAACCGTCTTTAC
r15	ATCGCCGTACGTTCCGCGAAAGTGCGGCGATTGTAGTTACGAGAGCGAATTTCCGGGAGG
r16	CGGTTTTGATAATATCTGCCGCAGAACCCTGGATCGGGGTGTTCATCGCCGTACGTTCCG
r17	CGCGGCGACGTTTGATCTCACGATGCAGGCGTACCATGGCGGTTTTGATAATATCTGCCG
r18	CAAAAATGAGTTCGTCGTGAACTTGGAGCAGCATACGAGACTTAACGCGGCGACGTTTGA
r19	CCAGATTTTTCATTTCTTCGAGTTCCTCTTCTGGAACTTCAAAAATGAGTTCGTCGTGAA
r20	ACGGAGAGCGGGACCGCTTGTTCCATAACCGTACGAACCAGATTTTTCATTTCTTCGAGT
r21	GCTTCATACCAAGTCTGACCGGTGTGGATATCAACTTTGAGCGGCACGGAGAGCGGGACC
r22	CTTCCTTTCGGGCTTTGTTAGCAGCCGGATCCTTATTTGGCTTCATACCAAGTCTGACC

**Table 2:** Oligonucleotides for the synthesis of *paz*

f4	TTTGTTTAACTTTAAGAAGGAGATATACATATGAGCCACCAGATCCGTTCTAAGAAAACCCTGTGGGAAC
f3	CTAAGAAAACCCTGTGGGAACTCGTTGGTCGTAATAAAGACGCGCTGCGTGATTTCCTGAAAGAACACCGTGGCA
f2	TGAAAGAACACCGTGGCACCATCCTGCTCCGTGACATCGCGTCTGAACACAAAGTTGTTTACAAACCGATCTTCA
f1	GTTGTTTACAAACCGATCTTCAAACGCTACAACGGTGACCCGGACCTGATCGAAGACAACTCTAACGACGTTGAA
r1	TTTTTTCAGTTCCGGGGTGTTCCAGTAACGTTCCAGGTGGTAGTCGTACCAGTGTTCAACGTCGTTAGAGTTGTC
r2	TTCGCCAGGATGATTGGTTGGTTCAGGTCAACCGGACCGAACTTTTTGTAGAACTCTTTTTTCAGTTCCGGGGTG
r3	ACAACGAACTGCGGCAGGAGGTGAACCAGGTCACCACGGTTGTGCTGACGCAGTGGTTTCGCCAGGATGATTGGT
r4	CTTCCTTTCGGGCTTTGTTAGCAGCCGGATCCTTAGTTATAAACCGGAACAACGAACTGCGGCA

### Gene synthesis

**Figure 1:**
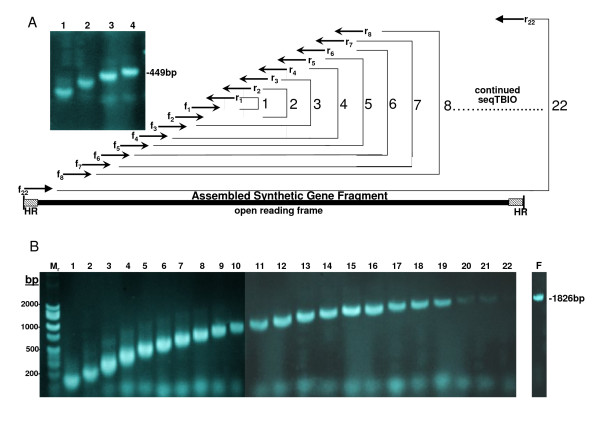
**SeqTBIO method of PCR-based gene synthesis for *paz *and *polA***. The nucleotide sequences *paz *and *polA *were assembled through a sequence of 4 and 22 reactions respectively and analyzed by agarose gel electrophoresis. Fragment sizes were compared against molecular markers ranging from 100 to 2000 bp. Two oligonucleotides were used for each amplification reaction starting with the central pair (f_1 _and r_1_), followed by the next pairs (f_2 _and r_2_, then f_3 _and r_3_, etc.) in the subsequent reactions, in an inside-out manner. The oligonucleotides overlapped by 15 to 23 nt. The entire *paz *sequence was synthesized in 4 reactions showing single products in each reaction with traces of unincorporated primers (A, lanes 1–4). The robustness of sequential step DNA assembly is shown for the synthesis of *polA *where single band fragments are clearly seen for each assembly step (B, lanes 1–22). Lane F shows the final synthesis product of *polA*. The assembled gene products for both proteins contained 30 nt of sequence at the 5'and 3' termini homologous to the terminal ends of a linearized plasmid vector of pET-3a for subsequent *in vivo *homologous recombination. These homologous regions (HR) were designed within the last outside primers sets used in the assembly process.

Each gene was assembled through a sequence of n reactions, where n is half the number of oligonucleotides used. Only 2 oligonucleotides were added to each reaction, starting with the central pair (f_1 _and r_1_), followed by the next pairs (f_2 _and r_2_, then f_3 _and r_3_, etc.) in the subsequent reactions, in an inside-out manner (Figure 1A). A total of 4 and 22 primer pairs were used for the synthesis of *paz *and *polA *respectively. Each reaction took place in a 12 μl volume and included Pfu Ultra II Fusion HS DNA polymerase (Stratagene, La Jolla, CA, USA) and its corresponding buffer according to the manufacturer's instructions, dNTP at a final concentration of 200 μM each, 0.9 ul of each oligonucleotide and 0.9 μl of the previous reaction product (0.9 μl distilled water instead in the first reaction). All PCR were performed in a Perkin Elmer GeneAmp PCR System 9600 thermal cycler. Each reaction was subjected to 7 cycles of 95°C denaturation for 15 s, 55°C annealing for 20 s and 72°C extension for 20 s. Extension time was increased to 30 s after the 12^th ^reaction and to 40 s after the 30^th^. Five μl of the last reaction were mixed with 50 ng of linearized pET3a and used for transformation.

### Transformation and sequence analysis

*E. coli *strain DH5α was rendered competent using the rubidium chloride method [5]. DNA mixtures were gently mixed with 50 μl competent cells. After 15 min incubation on ice, cells were heat-shocked at 42°C for 80 s. Thereafter, the transformed cells were diluted with 150 μl Luria-Bertani (LB) medium, incubated 1 hour at 37°C/250 RPM and spread on LB-agar plates containing 100 mg/L carbenicillin. Colonies were visible after 16 hours incubation at 37°C. Colonies were picked without screening and grown in 5–10 ml LB complemented with 100 mg/L carbenicillin at 37°C/250 RPM for 12–16 hours. Plasmids were purified using EZNA plasmid Miniprep kit II (Omega Bio-Tek, Doraville, GA, USA). Sequencing was performed by Functional Biosciences (Madison, WI, USA) using vector-specific sequencing primers flanking the cloning site as well as additional gene-specific primers when needed. Both strands of each gene were sequenced. Sequences were assembled and analyzed using the Staden package version 1.6.0 [6].

### Error correction

**Figure 2:**
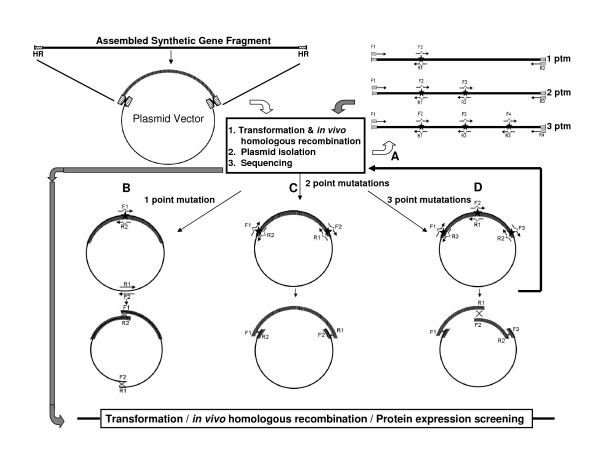
**Scheme of error correction using coupled methods of site directed mutagenesis and homologous recombination**. Assembled synthetic gene fragments subcloned into a plasmid vector are subjected initially to sequence analysis of the synthesized product to detect nucleotide errors. Error correction can be conducted in two ways using oligonucleotide primers (30–35 nucleotides long) that are designed to include the correcting nucleotide (when needed) at the midpoint. First, mutagenic primers are targeted exclusively against the assembled synthetic gene (route A). Two, three and four pairs of primers are required to correct one, two and three point mutation (ptm) sites respectively. DNA fragments are amplified by two or all primer pairs F1-R1, F2-R2, F3-R3 and F1-R4 in separate reactions. The terminal primers have overlapping homologous regions with that of the targeted plasmid vector. Reaction products are mixed for transformation into an appropriate cell host. The second approach involves the amplification of the plasmid vector (routes B-D). To remove 3 point mutations, two correcting primers, reverse-complement of each other, are designed at each mutation site, with the correcting nucleotide being at the midpoint of each primer. DNA fragments are amplified by PCR using primer pairs F1-R1, F2-R2 and F3-R3 respectively in 3 separate reactions (D). Two pairs of primers are similarly used for 2 point mutations involving only 2 separate reactions (C). Single site error correction requires a non-mutagenic primer pair corresponding to a sequence in the vector backbone in addition to the correcting primer set such that 2 fragments are generated (as if 2 corrections were being made). Products of the correcting reactions are retransformed into competent cells for plasmid isolation and sequencing. Upon verification of error free clones, the plasmids are then transformed into an appropriate host cell for protein expression.

Sequencing errors were mended by *in vivo *homologous recombination of overlapping plasmid fragments amplified by PCR. Oligonucleotide primers were 30–35 nucleotides long and were designed to include the correcting nucleotide (when needed) at the midpoint. The reverse primer of each overlapping fragment was the reverse-complement of the forward primer of the next fragment (Figure 2). Each fragment was amplified in a 20 μl reaction containing 2 μl (10×) PfuUltra II reaction buffer, 0.4 μl (10 mM each) dNTP, 0.4 μl PfuUltra II fusion HS DNA polymerase (Stratagene, La Jolla, CA, USA), 1 μl each appropriate (10 μM) forward and reverse primer and a variable amount of plasmid DNA template. The thermal cycling conditions of the PCR were as follows: initial denaturation at 95°C for 2 min; 25 cycles of 95°C for 20 s, 66–70°C for 20 s, 72°C for 90 s; final extension at 72°C for 2 min. When indicated, PCR products of the expected size were excised from a 0.8% agarose gel and purified using the QIAquick gel extraction kit (Qiagen, Valencia, CA, USA) according to the manufacturer's instructions. Unpurified PCR products were directly used for transformation without further manipulation. In both cases, 1 μl of each product were mixed and used to transform competent cells. Transformation, plasmid preparation and sequencing were performed as described above.

### Protein expression and purification

The two proteins were expressed and purified in exactly the same way unless indicated otherwise. Expression plasmids containing the error-free inserts were directly transformed into competent *E. coli *BL21(DE3). Competent cells were prepared by the rubidium chloride method and transformed with 10 ng of the recombinant plasmids as described previously. Transformed cells were then mixed with 150 μl LB medium, incubated 1 hour at 37°C/250 RPM and spread on LB-agar plates containing 100 mg/L carbenicillin. Overnight colonies were then used to inoculate 20 mL LB cultures containing 100 mg/L carbenicillin that were incubated for 8 hours at 37°C/250 RPM. The resulting cultures were further distributed into 2 × 2 L of LB medium containing the same type and amount of antibiotic. The cultures were allowed to grow at 37°C/250 RPM and the expression of the recombinant proteins was induced by the addition of isopropyl thio-β-D-galactoside (IPTG) at a final concentration of 0.5 mM when the optical density of the liquid culture at 600 nm reached 0.6.

After 6 hours, cells were harvested by centrifugation. All subsequent steps were performed at 4°C. Cells were resuspended in buffer A (50 mM TRIS pH 7.5, 50 mM NaCl, 1 mM EDTA for PolA and 50 mM BIS-TRIS pH 6.5 50 mM NaCl, 1 mM EDTA for PAZ) supplemented with 10 μg/mL deoxyribonuclease I and ribonuclease I (New England BioLabs, Ipswich, MA, USA) and disrupted by sonication (6 cycles of 45 pulses) using a Branson Sonifier 250 (VWR Scientific, West Chester, PA, USA). Cell debris were removed by centrifugation (12000 g, 20 min). The supernatant was heated for 30 min at 75°C and the precipitate was removed by further centrifugation. The supernatant was loaded into an ion exchange column (HiTrap Q sepharose for PolA and HiTrap SP for PAZ) (Amersham, USA) that had been pre-equilibrated with buffer A and eluted with a 0.05–1 M NaCl linear gradient in buffer B, using an Akta Explorer FPLC system (Amersham-Pharmacia, USA). Fractions containing the recombinant protein (showing a band of the expected size on a SDS-PAGE gel) were pooled, dialyzed against buffer A. The protein was concentrated to 2 mL using a Amicon Ultra centrifugal filter device (Millipore, USA), applied to a Sephacryl S-200 gel filtration column (Pharmacia, USA) pre-equilibrated with buffer A and eluted with the same buffer. Fractions corresponding to the principal peak were collected, pooled and concentrated for subsequent crystallization screening. The final concentration were 15 and 22 mg/mL for PolA and the PAZ domain respectively. Protein concentration was estimated using UV absorbance 280 nm and 260 nm and determined by the Bradford assay [7] using albumin as the protein standard.

### Gel Electrophoresis

Synthesized DNA products were analyzed by agarose gel electrophoresis as described by Sambrook & Russel [8]. Purified recombinant proteins were evaluated by SDS-PAGE analysis according to the method of Laemmli [9] using 12% polyacrylamide pre-cast gels (Invitrogen, Carlsbad, CA, USA) in the presence of 0.1% SDS. SeeBlue Plus2 Pre-stained standards (Invitrogen, Carlsbad, CA, US) were use as standard protein markers in the range of 4–250 kDa.

### Protein crystallization

Crystallization conditions for the purified proteins were screened with Crystal Screen I and II crystallization screening reagents (Hampton Research, Aliso Viejo, CA, USA) by sitting-drop vapor diffusion in IntelliPlates (Art Robbins, Sunnyvale, CA, USA). The final diffraction quality crystals were grown after approximately one week at room temperature from optimal conditions based around the initial conditions obtained from the commercial screen. The conditions for their growth will be reported in a separate publication with their structure determination.

### X-ray data analysis

Protein crystals obtained were visually observed through a polarized filter under a Nikon visible-light microscope and photographed with a Kodak Digital Science DC120 camera. The crystals were carefully soaked for 2 min in a cryopreservative solution containing 25% glycerol in precipitating reagent. Thereafter, the crystals were mounted onto a 20 micron diameter nylon cryoloop (Hampton Research, Aliso Viejo, CA) and directly flash frozen in liquid nitrogen. The cryogenically preserved crystals were mounted on a goniometer head cooled in a nitrogen stream at 100 K using an MSC X-Stream Cryogenic Crystal Cooler System (Molecular Structure Corporation, The Woodlands, Texas, USA). Diffraction data were collected using an MSC R-AXIS IV image plate detector with a crystal-to-detector distance of 250 mm. The X-rays were generated by a Rigaku rotating anode generator operated at 50 kV and 100 mA and focused with MSC OSMIC confocal mirrors. Images were collected at 1.0 degree oscillation angles with an exposure time of 5 min. These data were indexed using DENZO and reduced using SCALEPACK within the HKL2000 program package [10].

## Results

### Gene synthesis

Several methods for assembling genes from synthetic oligonucleotides have been developed [11-15]. Our effort to include gene synthesis in a gene to structure pipeline for non-automated structural genomics [16] prompted us to expand current methods in gene assembly for recombinant protein production. Our method is a modification on the thermodynamically balanced inside-out (TBIO) PCR-based gene synthesis [12] and is referred to as "Sequential TBIO (SeqTBIO)" because of the incremental and individual successive steps involved. The principle is illustrated in Figure 1. An initial DNA fragment made from 2 central oligonucleotides is extended bidirectionally, one oligonucleotide pair at a time. Major differences with the TBIO methods are the number of oligonucleotides per step (1 pair instead of 4 to 6), the number of cycles per step (only 7 compared to 25) and the absence of any gel purification step. Overall, despite a higher number of steps, our method is faster and more robust because the steps are simpler and require less manipulation.

To demonstrate the simplicity of our improved methodology for synthesizing DNA fragments of different sizes, the assembly of two novel nucleotide sequences, *paz *and *polA*, were performed. The protein encoded by *paz *is a 15.41 kDa PAZ (Piwi/Argonaute/Zwille) domain, a siRNA-binding domain of an Argonaute protein homologue. The *polA *gene codes for a deletion mutant of a family A DNA polymerase lacking the 5'-3' exonuclease domain, with a molecular weight of 67.70 kDa. The *paz *sequence was assembled in just 4 steps (Figure 1A). Each reaction resulted in a single band clearly showing an incremental increase in DNA fragment size. The assembled synthetic gene fragment contained a total of 449 base pairs and included vector homologous regions at its ends. Two clones were sequenced, one contained 2 point deletions and the other one was error-free and was subsequently used for protein expression.

Gene assembly results for *polA *are shown in Figure 1B. Each reaction involving the synthesis of *polA *resulted in a single band product. Because the yield began to decrease after about 20 reactions (reactions 20–22), the last reaction was repeated using more template (2 μl of the 21^st ^reaction product instead of 0.9) and more cycles (15 instead of 7), which was enough to obtain a pure single DNA product (lane F). The assembled synthetic gene fragment contained 1826 bp including sequences at the 5'and 3' termini that were homologous to the expression vector sequence. After co-transformation with a linearized vector and plasmid isolation from cultures grown from individual colonies, plasmids isolated from 4 clones were sequenced. All contained the expected insert, and among the 4 clones, a total of 6 single-nucleotide mutations were detected, including 1 deletion, 3 transitions and 2 transversions. A clone with a single mutation (the deletion) was selected for error correction.

### *In vivo *homologous recombination

We used *in vivo *homologous recombination to efficiently insert assembly products into a propagating plasmid vector and to rapidly correct synthesis errors. Gene cloning mediated by RecA-independent homologous recombination in *E. coli *is well documented [17-19] although it has not become a mainstream technique despite its simplicity and efficiency. It is based on the ability of many *E. coli *strains (including the RecA deficient ones used in cloning) to perform *in vivo *intermolecular recombination between DNA fragments sharing homologous sequences at their ends. In our experiments, synthetic gene fragments can be quickly subcloned into a linearized target plasmid vector (Figure 2) without restriction digest, ligation or other enzymatic manipulation. Virtually 100% of the resulting clones contained the correct insert, eliminating the need for screening. In addition, synthetic DNA fragments produced with our method can be easily assembled further into larger constructs by *in vivo *homologous recombination of overlapping fragments. We have successfully synthesized a 3.6 kb and a 6 kb genes by assembling two 1.8 kb and three 2 kb fragments respectively (data not shown).

### Errror correction by Site Directed Mutagenesis

Synthetic genes inherently have errors derived mainly from inaccuracies in the oligonucleotide syntheses and to a lesser extent from the DNA polymerase-mediated assembly [14]. The error rates observed in our assembly products were consistent with the 1 to 3 errors per kb reported by others [20], and imply that, especially for larger genes, prohibitively large numbers of sequencing reactions need to be performed in order to have a high probability to find a clone with the correct sequence [21]. Several approaches have been proposed to decrease the error rate, in particular using enzymes involved in mismatch recognition on renatured assembly products [20-22]. However, these may be difficult to implement due to the cost and availability of such enzymes. Therefore, for small-scale gene synthesis projects, it is often simpler to correct errors through site-directed mutagenesis (SDM). Numerous SDM techniques have been described over the last two decades, many of which involve more than one PCR step, the use of additional enzymes or further complex manipulations [23,24]. We applied a SDM method based on *in vivo *homologous recombination using a single PCR step, which is both simple and efficient [18,25]. In its simplest form, competent cells are directly transformed with PCR products from a single amplification step generating overlapping fragments.

The error correction strategy using *in vivo *homologous recombination is illustrated in Figure 2. We have approached it in two ways. First, primers are designed to produce corrected fragments of the gene assembly product (Figure 2A). In this case, two, three and four pairs of primers (F1-R1, F2-R2, F3-R3 and F4-R4) are required to correct one, two and three error sites respectively in separate reactions. The resulting PCR products are mixed with the linearized plasmid vector and used to transform competent cells in which *in vivo *homologous recombination is allowed to occur. In our hands, as many as 4 PCR amplified corrected fragments recombined accurately with the recipient vector and generated error corrected products.

The second approach involves the amplification of DNA fragments that include the plasmid vector (Figure 2, panels B-D). Typically, up to 3 point mutations are corrected at one time. In such a case, two correcting primers, reverse-complement of each other, are designed at each mutation site, with the correcting nucleotide being at the center of each primer. DNA fragments are amplified by PCR using primer pairs F1-R1, F2-R2 and F3-R3 respectively in 3 separate reactions. In the case of 2 point mutations, two pairs of primers are similarly used such that there will be only 2 separate reactions. When a single synthesis error needs to be corrected, a non-mutagenic primer set corresponding to a sequence in the vector backbone is used in addition to the correcting primer set such that 2 fragments are generated (as if 2 corrections were being made) in order to avoid using mutually annealing primers in a single reaction.

In correcting the gene synthesis error of *polA *that had a point deletion, the two strategies were pursued in parallel. First, 2 overlapping fragments of the same plasmid were amplified (illustrated in Figure 2, panel B), each with a primer correcting the deletion and a primer corresponding to a sequence in the vector backbone. The primers in each fragment were reverse complement of the primers in the other fragment. In the second approach, only the gene synthesis product was amplified from the plasmid template in 2 fragments (each fragment being amplified with a correcting primer and a terminal oligonucleotide used for the original gene synthesis). In this case, the amplification products were mixed with the linearized vector allowing *in vivo *recombination to occur between the 3 fragments (illustrated in Figure 2, panel A). In both cases, 40 pg/μl of plasmid template was used in the correcting PCR mixture. Between 10 and 100 colonies were obtained for each transformation.

A random selection of colonies was analyzed for successful recombinant inserts. In both correction procedures used, the correction efficiency was 50% in which half the clones analyzed contained completely accurate sequences of the recombinant insert. A corrected clone from the first approach was selected for protein expression to demonstrate the integrity of the plasmid in the face of possible site mutations in the vector sequence.

An obvious concern when plasmids are used as PCR templates is the risk that they compete with the desired recombination products after transformation. Even if amplified linear DNA is several orders of magnitude more concentrated than the circular plasmid template in the PCR products, homologous recombination being a rare event, even a modest amount of circular plasmid template may result in the unwanted presence of non-recombined clones among the colonies after transformation. Several strategies have been proposed to address this problem, including gel purification of the amplified DNA fragment, linearization of the plasmid template by restriction enzyme digestion prior to PCR [18] and treatment of PCR products with DpnI which cleaves methylated DNA [26]. In our effort to minimize the number of steps and to simplify the method, we attempted to prevent plasmid carry-over by diluting the plasmid template in the correcting PCR. In the case of *polA*, this strategy succeeded but the dilution level was not optimal since only half of the clones were recombination products.

When applying the same error correction technique to other genes, we found that further diluting the template to 3 pg/μl or less in the correcting PCR consistently resulted in 100% correction efficiency, effectively decreasing template carry-over to negligible levels. However, there were unexpected consequences as new mutations appeared in otherwise corrected clones. Despite using one of the highest-fidelity DNA polymerases commercially available, unintended mutations appeared when plasmid templates were diluted to 10 pg/μl or less. The mutation rate seemed to correlate with the level of template dilution, with over 1 mutation per kb when the template concentration was under 1 pg/μl. All new mutations involved a single nucleotide. A compilation of 79 mutations detected after sequencing 94 kb of corrected clones showed transitions to be prevalent (59% of all mutations observed) with an equal amount of type 1 (A to G and T to C), and type 2 (G to A and C to T), followed by deletions (28%), transversions (9%) and insertions (4%). Note that in all cases where new mutations were observed the template concentration was significantly lower than the DNA polymerase manufacturer's recommended 100 to 600 pg/μl of plasmid template input. In order to obtain reproducible 100% error correction while avoiding additional mutations, we found it necessary to limit the template dilution in the correcting PCR to the levels recommended by the enzyme's manufacturer and therefore to include a template removal step before transformation.

Our observations of increased mutation rates when using highly diluted templates in PCR are consistent with a previous report that low copy number template can decrease the fidelity of both Taq and Pfu DNA polymerases [27]. To our knowledge, no explanation was ever proposed to account for such a phenomenon. However, it can have serious implications for PCR methods involving samples with limited availability such as ancient DNA, forensics or pre-implantation genetic diagnosis. Further studies are needed to confirm whether this phenomenon affects PCR in general and how the sensitivity to template concentration varies among different DNA polymerases or with varying reaction conditions.

### Protein expression, crystallization and preliminary X-ray analysis

**Figure 3:**
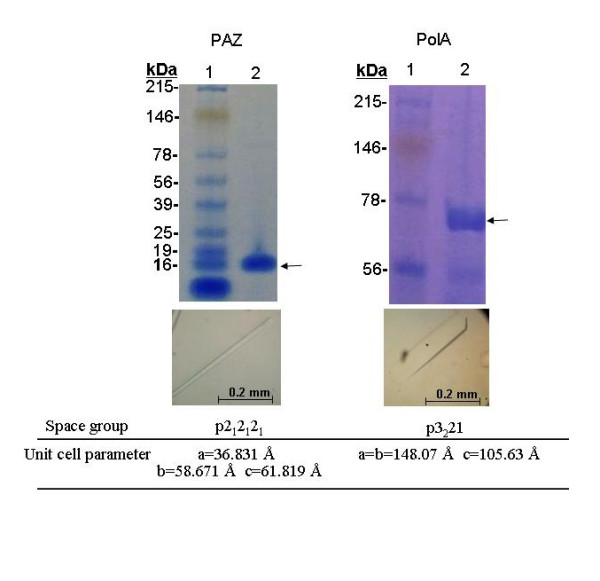
**SDS-PAGE analysis and crystallization of PAZ and PolA**. Purified recombinant proteins PAZ and PolA are shown in the upper panels (lanes 2) against standard molecular markers (lanes 1) containing myosin, phosphorylase, BSA, glutamic dehydrogenase, alcohol dehydrogenase, carbonic anhydrase, myoglobin red, lysozyme, aprotinin and insulin B chain on a 11% polyacrylamide gel. Approximately 15 μg of purified protein was loaded in each gel (lanes 2) and visualized with Coomassie brilliant blue. The arrows indicate recombinant proteins at about 15 kDa and 70 kDa for PAZ and PolA respectively. The proteins were estimated to be more than 90% homogeneous. Crystals of PAZ and PolA are shown in the bottom panels along with their corresponding space group and unit cell parameters as determined by preliminary X-ray analysis.

Expression of recombinant proteins PAZ and PolA were performed by transforming an *E. coli *expression cell strain with the plasmids containing the error-free inserts. A random selection of resulting colonies was screened for small scale expression. Clones that demonstrated recombinant expression as determined by SDS-PAGE analysis were further cultured for large scale preparation. The yields of both proteins were approximately 30 mg/L. Since both PAZ and PolA recombinant proteins were derived from hyperthermophilic microorganisms, they had the outstanding property of being heat resistant. Thus, the proteins can be immediately purified to more than 70% after a heat selection step. PAZ and PolA can be purified to more than 90% homogeneity based on SDS-PAGE analysis after ion exchange and size exclusion chromatography (Figure 3). The approximate size of the purified proteins were 15 kDa and 70 kDa for PAZ and PolA respectively. In both cases, protein crystals suitable for X-ray diffraction were obtained within 5 days, and as a result their space group and unit parameters were determined. PAZ crystals appeared as orthorhombic habit and grew as large as 0.5 mm in the longest dimension. The space group of the crystals was determined to be P2_1_2_1_2_1 _with unit cell dimensions of a = 36.831 Å, b = 58.671 Å and c = 61.819 Å. Prismatic crystals of PolA grew as big as 0.4 mm in the longest dimension. The space group and unit cell dimensions were identified to be P3_2_21 and a = b = 148.07 Å, c = 105.63 Å respectively. The crystals of PAZ and PolA diffracted in the range between 2.5–8 Å resolution using a home laboratory X-ray source. The crystallographic structure determination of both proteins is in progress.

## Discussion

**Figure 4:**
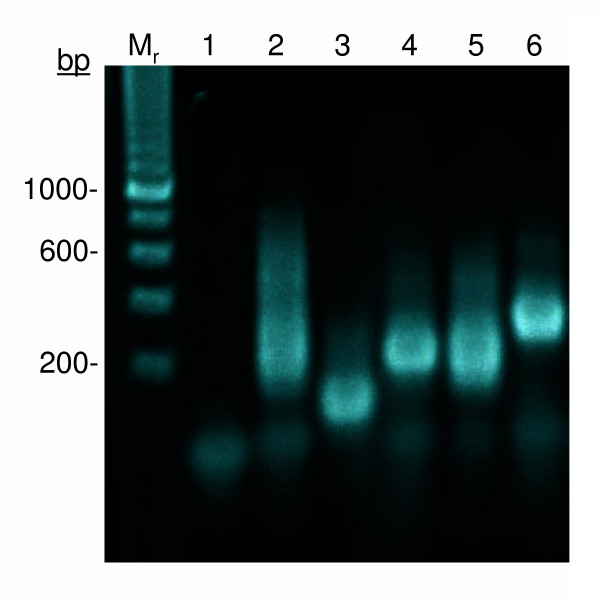
**Comparative gene synthesis of polA coding region by methods of TBIO and SeqTBIO**. The oligonucleotide pairs f_1_-r_1_, f_2_-r_2_, f_3_-r_3 _and f_4_-r_4 _were used to attempt the synthesis of a 351 bp central fragment of the polA coding region. The initial TBIO gene synthesis included the mixture of 8 primers and the reaction was subjected to 30 cycles of PCR amplification (lane 2). An early version of the SeqTBIO approach was performed in parallel (lanes 3–6) using the first 3 oligonucleotide pairs only. The central pair (f_1_-r_1_) was initially assembled (lane 3) followed by next pairs f_2_-r_2 _(lane 4, 5) and f3-r3 (lane 6). The TBIO reaction produced a smear and did not seem to generate a unique fragment but instead a range of product sizes ranging from 190 bp – 600 bp. In contrast, each SeqTBIO reaction generated a single-band product of the expected size. A PCR negative control is shown in lane 1. The reaction products were analyzed by electrophoresis on a 1.2% agarose gel stained with ethidium bromide and visualized by UV illumination.

The approach to DNA synthesis described here was specifically designed for rapidly assembling DNA coding regions for crystallizing recombinant proteins. The sequential method, seqTBIO, builds one pair of oligonucleotide fragments at a time by PCR-based gene synthesis resulting in reliable and consistent DNA assembly for almost any sequence. In standard PCR-based gene synthesis, 4–6 primer pairs are used for amplification at a time. Thus in most cases secondary products are easily produced from non-specific amplification requiring purification after each assembly cycle. The integrity of the gene synthesis also cannot be readily monitored after an assembly of each oligonucleotide set with batch PCR-based synthesis protocols. We have compared the synthesis of *polA*, along with other genes not reported here using established PCR-based gene synthesis procedures [12,15,28,29]. The resulting gene assemblies were problematic (Figure 4) requiring extensive optimization of the PCR conditions in each step. This is particularly true for larger gene fragments (greater than 1 kb). The reactions often produced non-specific or multiple gene products as revealed by numerous or smeared bands in gel electrophoresis analysis. Even though our approach is not guaranteed to be fail-safe, it has shown to have great consistency in the continuous synthesis of homogeneous DNA products for different types of gene sequences throughout the assembly steps.

Error corrections have been the limiting factor to completing accurate gene synthesis. We have corrected sequence errors using site-directed mutagenesis coupled with homologous recombination [18,25] because of its simplicity and efficiency. Two tactics have been considered and both offer high-speed repair and subcloning independent of restriction site availability and ligation reactions. One method involves the amplification of an entire circular plasmid by PCR using mutagenic primers with overlapping sequences, while the other approach utilizes PCR amplification with mutagenic primers against the assembled synthetic gene fragment only. Even though we have demonstrated successful protein expression with corrected constructs using the former, we have observed higher success in subsequent protein expression trials with the latter approach. This is because the full-length amplification of the plasmid can give rise to second-site mutations in the vector sequence. Consequently, the expression plasmid may be rendered dysfunctional in replication, transcription or translation diminishing the occurrence of transformed cells that would be able to overexpress the targeted gene product. However, most of the time, the error corrected inserts can be subcloned into another vector. In the case of correcting errors by using mutagenic primers to amplify the assembled synthetic gene fragment only, there is minimal concern in altering the expression vector sequence since no part of the plasmid is amplified. As a result, the plasmid can be used for protein expression with increased confidence that possible failure does not result from alterations in the vector sequence.

Coupling a more reliable DNA synthesis approach with an efficient means of error corrections without any additional purification or enzymatic requirements provides a rapid means to clone and express proteins when the source of genomic or complementary DNA is limited or not available at all. This is particularly useful in crystallographic studies where altering specific amino acid sites or regions is required for overexpression or structural packing. Protein crystallization is often very sensitive to inter- and intramolecular packing where, for example, producing optimal ionic network interfaces through the apposition of extensive hydrophobic surfaces [30] or even constructing protein chimeras determine the successful outcome of a crystallographic structure [31]. In addition, strategic incorporation of sulphur [32,33] containing residues or methionine for selenomethionine replacement would be very useful in constructing protein molecules with intrinsic atoms useful for crystallographic phasing (i.e. single-wavelength-anomalous diffraction) [34].

**Figure 5:**
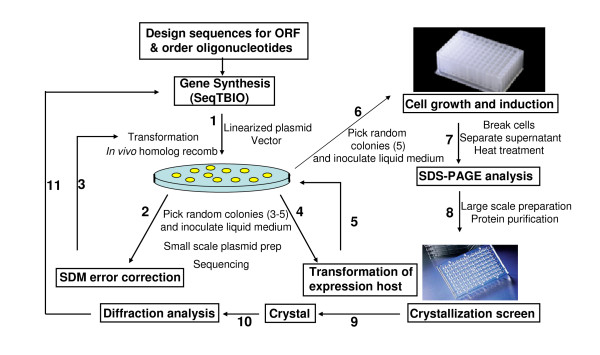
**Gene to crystal flowchart**. Gene synthesis can be integrated into an experimental pipeline where proteins can be designed for crystallization screening. It is possible to design variations of protein constructs by exploiting the SeqTBIO method of sequential DNA assembly (path 1 to 6). Different constructs can be screened for expression in 96-well format array and evaluated for the protein solubility and stability (path 7). In our case, we used heat stability as a biochemical selection criterion. However, recombinant tags can be easily incorporated at the DNA synthesis level whereby the resulting expressed proteins can be selected with affinity binding matrices (e.g. His or GST tags). Large scale production can then be performed for crystallization trials (path 8–9). Initial crystals obtained would undergo optimization and preliminary X-ray analysis (path 10). Further protein sequence changes can be made if necessary for further processing (path 11).

It is very feasible to integrate gene synthesis into a non-automated gene to crystal pipeline such that genes can be systematically designed, synthesized and implemented for recombinant expression followed by crystallization screening in a efficient throughput manner. Figure 5 illustrates the flow of execution from design to crystal. Once the sequence of the open reading frame has been decided, the process from ordering the oligonucleotides to crystallization screens can be realized within 2 weeks. One of the most time consuming steps found in structural genomics pipelines at the level of protein expression is subcloning the amplified products into appropriate expression vectors by restriction digest and ligation reactions followed by transformation into a propagating host [16]. We have reported directional cloning methods that use *in vivo *homologous recombination to be the most efficient and cost effective because cloning can be accomplished without purchasing special vectors or expensive enzymes. The procedure can be achieved rapidly using multichannel pipettes to dispense multiple reactions and bulk transformation in competent cells. We have routinely obtained over 95% success in recombinant insertion of the synthesized or modified gene fragments. We have been using the pET vectors (Novagen, Madison, WI, USA) for successful expression of hyperthermophilic proteins. No tagging was necessary since a heat selection step was included as part of the initial purification procedure and used as an indicator for thermal stability and solubility. In high throughput mode, it is possible to examine multiple synthesis products and undergo expression trials in 96-well plates as reviewed by Hughes and Ng [16].

The *paz *and *polA *genes are shown here as examples on how we have used the gene synthesis technique to produce novel proteins for protein crystallization. When the natural coding sequences of both proteins were cloned and prepared for recombinant protein expression in *E. coli*, all attempts to produce proteins failed. Both sequences showed codon usage that significantly diverged from that of *E. coli*. Recombinant expression could only be obtained after codons were optimized.

In our pipeline, large scale expression routinely involves two chromatographic steps executed after an initial heat selection. This includes separation by ion exchange and size exclusion chromatography and the proteins are usually screened immediately for crystallization. In the case of PAZ and PolA, crystals were obtained in the initial screening conditions and subjected to preliminary X-ray analysis. General crystallization conditions can be optimized in many ways and have been discussed previously [16,35,36]. If proteins crystallize poorly or not at all after exhaustive searches in crystallization conditions, then changing the protein molecule itself would be reasonable. Molecular changes can be made quite readily at the synthesis level where strategic amino acid residues or domains can be rearranged at will to optimize molecular packing. One interesting possibility, although untried, is to attempt expressing the proteins in incremental fragments that corresponds to the inside-out direction of synthesis of the recombinant open reading frame. Since gene synthesis can be incremental, each assembly cycle can be subcloned and tried for protein expression and crystallization. This possible approach would be of great interest in examining the truncation effects of the N- and C-termini of a protein on molecular stability, packing and ultimately crystallization. Other variations can involve domain switching or elimination and symmetry imposition. There are obviously countless other possibilities by which incremental changes of protein fragments may give rise to novel assemblies. The gene synthesis technique reported here integrates well into a gene to crystal pipeline, and its approach is applicable to small structural genomics projects as well as protein engineering studies.

## Conclusion

The SeqTBIO method of gene synthesis, associated with *in vivo *homologous recombination-mediated cloning and error correction, was shown to integrate well in a gene to structure pipeline. As illustrated by the examples of the *paz *and *polA *genes, gene assembly was simple, efficient, and did not require any specific reagent or enzyme other than those used in PCR, nor any complex manipulation. Cloning and error correction were straightforward, requiring nothing more than transforming competent cells with amplified gene fragments. The described methodology to generate cloned genes of any sequence for protein expression and further structural studies does not require any expensive equipment or particular technical skill and can therefore benefit laboratories with limited resources.

## Authors' contributions

DM designed and carried out gene synthesis, cloning and error correction experiments. RCH and MLBS carried out expression, purification and crystallization of PAZ and PolA respectively. JDN coordinated the study. DM and JDN co-drafted the manuscript. All authors participated in the revision of the manuscript and read and approved the final version.
